# Genome editing of *DWARF* and *SELF-PRUNING* rapidly confers traits suitable for plant factories while retaining useful traits in tomato

**DOI:** 10.1270/jsbbs.23063

**Published:** 2024-04-04

**Authors:** Ai Nagamine, Hiroshi Ezura

**Affiliations:** 1 Institute of Life and Environmental Sciences, University of Tsukuba, 1-1-1 Tennnodai, Tsukuba, Ibaraki 305-8572, Japan

**Keywords:** tomato, genome editing, plant factory, CRISPR–Cas9, *DWARF* (*D*), *SELF-PRUNING* (*SP*), GABA

## Abstract

Plant factories with artificial light are less affected than open-air areas to environmental factors in crop cultivation and are attracting attention as one of the solutions to the world’s food problems. However, the cost of cultivation in plant factories is higher than open-air cultivation, and currently, profitable factory-grown crop varieties are limited to those that are small or have a short growing period. Tomatoes are one of the main crops consumed around the world, but due to their large plant height and width, they are not yet suitable for mass production in plant factories. In this study, the *DWARF* (*D*) and *SELF-PRUNING* (*SP*) genes of the GABA hyperaccumulating tomato variety #87-17 were genome-edited by the CRISPR–Cas9 method to produce dwarf tomato plants. The desired traits were obtained in the T_1_ genome-edited generation, and the fruit traits were almost the same as those of the original variety. On the other hand, the F_2_ cross between #87-17 and Micro-Tom containing the *d* and *sp* mutations was dwarfed, but the fruit phenotype was a mixture of the traits of the two varieties. This indicates that genome editing of these two genes using CRISPR–Cas9 can efficiently impart traits suitable for plant factory cultivation while retaining the useful traits of the original cultivar.

## Introduction

Plant factories with artificial light are less susceptible to environmental influences in crop cultivation and are attracting attention as one of the solutions to coping with recent environmental changes, expanding cultivation in areas unsuitable for farming and providing sustainable agricultural technologies. To date, many companies have taken up the challenge of growing crops using plant factories and have gained experience with lettuce, herbs, strawberries, tomatoes, etc., and the market distribution of leaf lettuce and small leafy vegetables in particular is expanding (Kalantari *et al.* 2017, Nijwala 2021). Since these crops are often grown for fresh consumption, there is a certain amount of added value in crops grown in a thoroughly controlled and clean environment, such as a plant factory, since quality loss due to insect damage can significantly affect product value. Thus, if the value of the cultivated crop can be deducted from the cost of cultivation, vertical farming conducted in plant factories could become an even more commercially viable agricultural method. However, the equipment and operating costs associated with cultivation are added to the price of plant factory-produced crops. In fact, a survey of Japanese consumers showed that they felt plant-farm crops were “sanitary but expensive” (Kurihara *et al.* 2014). Moreover, although consumers are willing to pay up to 20–40% more for crops grown in plant factories than for crops grown outdoors, they still appear to prefer outdoor crops (Kurihara *et al.* 2014). Similarly, in Taiwan, it was suggested that whether the price is comparable to that of open-field cultivation affects the willingness to purchase (Huang 2019). To change this consumer mindset, it is imperative to reduce production costs and develop value-added products.

Thus, at present, several conditions must be met for a crop to be commercially suitable for vertical farming in plant factories. First, the plants must be small: according to a 2013 article by T. Kozai, the maximum size of crops suitable for a plant factory with 40-cm tall tiers, for example, is currently up to 30 cm (Kozai 2013). This is to ensure that the number of tiers in the plant factory is sufficient to maximize the efficient use of the limited cultivation space. Second, it is also important to have a short growing period to harvest, i.e., a greater annual growing cycle, and added value (e.g., no blemishes, low growing losses, etc.) commensurate with the cost of cultivation (Kozai 2013). Leaf lettuce and other leafy vegetables meet these requirements the best, and the most commonly grown plant factory crops today are also leafy vegetables such as leaf lettuce and herbs (Kalantari *et al.* 2017). Therefore, if these cultivation characteristics can be introduced into cultivars that are currently considered unsuitable for plant factories, it will be possible to produce new crops suitable for mass production in plant factories.

Tomato (*Solanum lycopersicum* L.) originates from the Andes region of South America, and its cultivation as a crop began in the 18th century. It is the most widely cultivated vegetable in the world today. To date, in addition to tomatoes produced through open-air cultivation, tomatoes produced in solar-powered plant factories have also been available on the market, allowing the application of semihydroponic cultivation. However, commercial cultivation in a fully enclosed plant factory with artificial light remains a challenge. This is mainly because the size of the plants is not suitable for a plant factory and the cost of long-term cultivation for harvesting the edible fruits is too high. On the other hand, there is a dwarf tomato variety, cv. Micro-Tom, which is not distributed as an edible tomato but can be grown stably in plant factories.

Micro-Tom, a dwarf tomato cultivar, has been widely used in molecular biology studies of tomatoes because of its compact growth characteristics; Micro-Tom not only has a low plant height of approximately 15 cm but also has small leaves and fruits, enabling the cultivation of a large number of plants in a small space. It also grows normally under fluorescent light, and the time from sowing to fruiting is short, with fruits in approximately 3 months. At least three genes, *DWARF* (*D*: Solyc02g089160), *SELF-PRUNING* (*SP*: Solyc06g074350) and *miniature*, have been implicated in dwarf traits among these characteristics (Bishop *et al.* 1996, Marti *et al.* 2006, Pnueli *et al.* 1998). Although the *miniature* gene is still unidentified, Micro-Tom’s contribution of this gene to dwarf traits is not much higher than that of the other two genes (Pnueli *et al.* 1998). Therefore, if we can establish a method to introduce the Micro-Tom dwarf trait into currently available tomato varieties by introducing mutations in two genes, *DWARF* and *SELF-PRUNING*, it will be possible to modify any variety into a variety suitable for plant factories.

To impart a specific trait to a given variety, traditional cross-breeding techniques have long been used. However, this method requires a great deal of labor, such as repeated backcrossing, to remove traits other than the specific trait of interest. In addition, depending on the genomic location, it may be virtually impossible to separate the target trait from the unfavorable trait by crossbreeding. Since the publication of the first paper on CRISPR-Cas9 by Charpentier and Doudna in 2012 (Jinek *et al.* 2012), an explosion of research and applications of the CRISPR–Cas9 method of genome editing have been underway to address these traditional problems in crossbreeding and breeding. The usefulness of this method is evident in the fact that only nine years after its discovery, the first genome-edited crop produced by CRISPR–Cas9 was launched, and it is now more than just a single precious example (Ezura 2022).

In this study, a GABA hyperaccumulator genome-edited tomato cultivar, #87-17, which lacks the autoinhibitory domain of the *SlGAD3* gene (Lee *et al.* 2018, Nonaka *et al.* 2017), was endowed with traits suitable for plant factory cultivation, creating a system to produce crops adapted to plant factory cultivation more rapidly than conventional crosses. The *DWARF* and *SELF-PRUNING* genes in #87-17 were genome edited with CRISPR–Cas9, and the dwarf and fruit traits of the T_1_ lines produced were evaluated and simultaneously compared with those of the *d*/*sp*/*GAD3* triple mutants produced by conventional breeding methods.

## Materials and Methods

### Materials

Tomato (*Solanum lycopersicum* L.) ‘Micro-Tom’ seeds were provided by the National Bioresource Project (Saito *et al.* 2011). The seeds of #87-17 and the original cultivar cv. Sicilian Rouge CF were provided by Sanatech Seed Co. (Tokyo, Japan).

### Guide RNA selections

Guide RNAs for genome editing of *DWARF* and *SELF-PRUNING* genes were selected from the candidates predicted by CRISPR-P v2.0 (http://crispr.hzau.edu.cn/CRISPR2/) with an On-Score of 0.5 or higher, low predicted off-target, G/C content of 30–80%, and a guide RNA secondary structure score within the acceptable range were selected as appropriate. The sequence of each guide RNA used is shown in the Supplemental Table 1.

### Vector construction and tomato transformation

The vector pDeCas9-Kan (Fauser *et al.* 2014, Mikami *et al.* 2015) was used for genome editing of the *DWARF* and *SELF-PRUNING* genes by CRISPR–Cas9. The modification of these plasmids was carried out following the method presented in previous studies (Fauser *et al.* 2014, Mikami *et al.* 2015). The constructed vectors were introduced into *Agrobacterium tumefaciens* GV2260 *via* electroporation. Tomato transformation was carried out with the tomato-optimized protocol (Sun *et al.* 2006), and then the regenerated diploid shoots that were rooted under the selection media (1/2 MS medium containing 1.5% (w/v) sucrose, 50 μg/mL kanamycin, 375 μg/mL Augmentin, and 0.3% (w/v) Gelrite, pH 5.6–5.8) were selected as T_0_ plants.

### Plant cultivation

The selected T_0_ plants were grown in a culture room at 25°C under fluorescent light (300 μmol/m^2^/s) in a 16:8 hr light:dark photoperiod until the respective indels were confirmed and T_1_ seeds were obtained. During cultivation, a standard nutrient solution (Otsuka A; Otsuka Chemical Co., Ltd., Osaka, Japan) was supplied. Harvested T_1_ seeds were seeded and transplanted on rockwools (5 × 5 × 5 cm) and then cultivated under the same conditions after indel checking. Plant measurements were carried out at 30, 55, 95 and 123 days after transplant (DAT), and then red-ripe fruits were harvested. The plants that were used for Micro-Tom × #87-17 crossing and the F_1_ and F_2_ plants were also cultivated under the same conditions. Plant measurement for the F_2_ triple mutants was carried out at 123 DAT, and then red-ripe fruits were harvested.

### Indel detection

For indel detection, PCR fragments were specifically amplified using target site-specific primers (Supplemental Table 1) against genomic DNA extracted from each transformant leaf and then sequenced using the same primers.

### F_2_ production by crossing #87-17 with Micro-Tom

After removing the anthers from the unflowered buds of Micro-Tom cultivated under the growing conditions described in the Plant cultivation section, pollen from #87-17 was used to pollinate the Micro-Tom buds. The resulting F_1_ seeds were then self-crossed to obtain F_2_ seeds. The resulting F_2_ seeds were sown, genomic DNA was extracted from each shoot, and triple mutants were selected using their respective dCAPS markers (Supplemental Table 1) and CAPS markers for *SlGAD3* mutations derived from #87-17 (Lee *et al.* 2018).

### Plant measurement

The height and width of the plants were measured at 30, 55, 95, and 123 DAT. The number of days from transplanting to first flowering was also measured. Yield per plant was recorded at approximately 123 DAT for Micro-Tom and up to the cultivation end for the other lines. The maximum vertical (A) and horizontal (B) fruit size was measured with calipers for each fruit. The fresh weight of each fruit was measured then seeds were collected for the next generation of cultivation. After the seeds were removed, each fruit was frozen in liquid nitrogen and stored in a deep freezer at –80°C until the time of GABA content analysis.

### Fruit GABA content measurement

The frozen fruit samples stored in a deep freezer were crushed by mixing with tap water (9 times the weight of the fruit) for 30 seconds in a blender, and then the stirred samples were filtered through a coffee filter to obtain GABA-containing samples. Samples obtained from fruit #87-17 were diluted 2-fold with tap water prior to analysis to keep values within the kit’s measurement limits. The GABA assay was performed by an enzymatic method according to the GABA-Miel^®^ (ENZYME SENSOR, Ibaraki, Japan) manual.

### PCA

PCA was performed on the fruit measurement data in R, and the PCA results were visualized using the ggbiplot package.

### Statistical analysis

Each measurement was compared using the Tukey–Kramer test. Data are expressed as the means ± S.D.s, swarm plot or box plots. The Tukey–Kramer test was used to detect significant differences between each experimental group.

## Results

### Genome editing of DWARF and SELF-PRUNING genes using CRISPR–Cas9

**Fig. 1.**
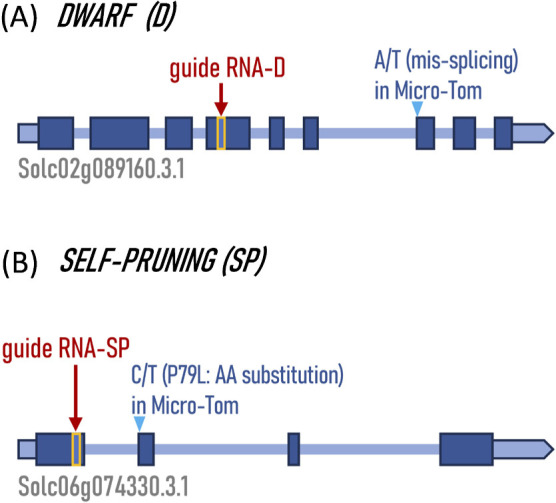
The gene structure of *DWARF* and *SELF-PRUNING* and the guide RNA sites for CRISPR–Cas9 genome editing. (A) *DWARF* (Solc02g089160) gene structure. SNP in cv. Micro-Tom (Marti *et al.* 2006) is shown with the pale blue arrow head with SNP detail. (B) *SELF-PRUNING* (Solc06g074330) gene structure. SNP in Micro-Tom (Pnueli *et al.* 1998) is shown with the pale blue arrow head with SNP detail. Each dark blue box shows an exon or UTR, and each light blue line shows an intron. Guide RNA sites for each gene are highlighted with yellow boxes and red arrows.

We designed guide RNAs for each gene with the goal of simultaneously losing the function of *DWARF* (*D*) and *SELF-PRUNING* (*SP*), two genes proven to be involved in Micro-Tom dwarfism, to confer Micro-Tom dwarfism to #87-17. The guide RNAs for each gene were designed with the goal of simultaneously inducing functional loss of the two genes *DWARF* and *SELF-PRUNING*. For this purpose, CRISPR-P v2.0 was used to narrow down the candidates to those that had the lowest possible off-target potential, met the recommended criteria and were located as far upstream of the genes as possible. The location of the guide RNAs in each gene is shown in Fig. 1, and sequence information is shown in the Supplemental Table 1. The guide RNAs for each gene were recombined into a single binary vector containing CRISPR–Cas9 and then transformed *via* Agrobacterium into 400 cotyledones each of #87-17 and the original variety from which it was derived, Sicilian Rouge CF (SR).

Among the obtained T_0_ population, the plants that were diploid and contained indels were screened, and 16 and 19 lines of T_0_ plants derived from #87-17 and SR, respectively, were selected. After each T_0_ line was grown, T_1_ seeds were collected from the resulting fruit, and approximately one week after T_1_ transplant, T_1_ plants showing the dwarf phenotype were selected, and the indels of each T_1_ line were confirmed by sequencing (Table 1).

**Fig. 2.**
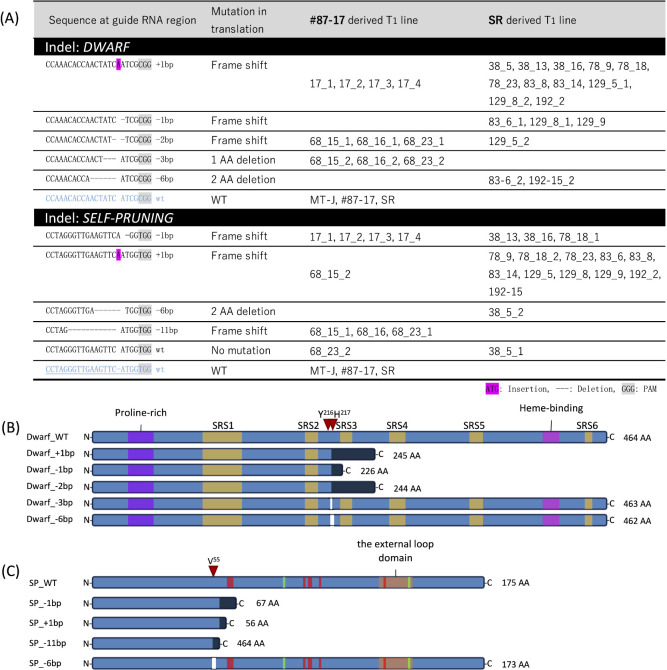
Indels of T_1_ lines. (A) List of indels for each of the *DWARF* and *SELF-PRUNING* genes in the T_1_ lines. (B) Schematic of the mutation pattern of the DWARF protein; the conserved and functional domains of the DWARF protein are highlighted in colored boxes (Kim *et al.* 2005). (C) Schematic of the mutation pattern of the SELF-PRUNING protein by each indel, showing residues that bind to the 14-3-3 protein (red boxes) and those that provide cues for florigen/antiflorigen function (green boxes) (Kang *et al.* 2022). Red arrows indicate the first deleted amino acid in the protein. Black indicates amino acid sequence changes due to frameshifts. AA: amino acid, SRS: substrate recognition sites.

Three individual T_1_ lines derived from the same T_0_ line were secured whenever possible. Indel results for the T_1_ lines selected in this way are shown in Fig. 2A. For both genes, genome editing of +1, –1, –2, and –11 bp that could induce early translation termination by frameshift occurred in many T_1_ lines, while genome editing of –3 and –6 bp that induced only a loss of 1 or 2 amino acids also occurred in some T_1_ lines (Fig. 2A, 2B). These deletions of –3 and –6 bp of the *DWARF* gene did not occur in known conserved domains (Fig. 2B).

### Observation of differences between genome editing and crossbreeding in conferring dwarfism on #87-17 and SR

**Fig. 3.**
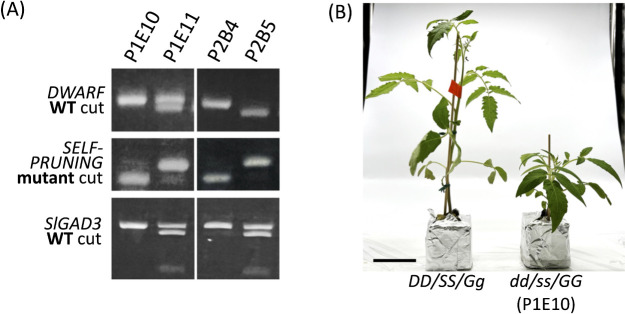
F_2_ triple mutant screening from F_2_ segregants. (A) Genotyping result of each F_2_ by dCAPs (*d* and *sp*) and CAPs (*SlGAD3*). (B) Side view of F_2_ triple mutant plant 14 DAT. The left plant is a control that does not contain any *d* or *sp* mutation. *GAD3* lacking the autoinhibitory domain is dominant against wild type therefore is indicated by capital letters. Bar = 5 cm.

To confer the dwarf trait on #87-17 using conventional breeding methods, we crossed #87-17 with Micro-Tom, and from 192 plants of the F_2_ segregating population, we selected two triple mutants, P1E10 and P2B4, containing the Micro-Tom alleles *d* and *sp* of *DWARF* and *SELF-PRUNING* and the self-repression domain deficient *SlGAD3* (*GAD3*) gene of #87-17. For *d* and *sp*, we designed dCAPS (sequence information is provided in the Supplemental Table 1), and for *GAD3*, we used the CAPS from Lee *et al.* 2018 (Fig. 3A). Approximately two weeks after transplanting to rockwool, the F_2_-triple mutants already showed a dwarfing trend, as shown in Fig. 3B. Looking at other F_2_ segregants of the same age, dwarfing was observed only when *d* was homozygous with respect to plant height, and no dwarfing was observed when *sp* was homozygous but *d* was not (Supplemental Fig. 1). This suggested that *sp* has no significant effect on plant dwarfing, which is consistent with previous reports (Bishop *et al.* 1996, Marti *et al.* 2006, Pnueli *et al.* 1998).

**Fig. 4.**
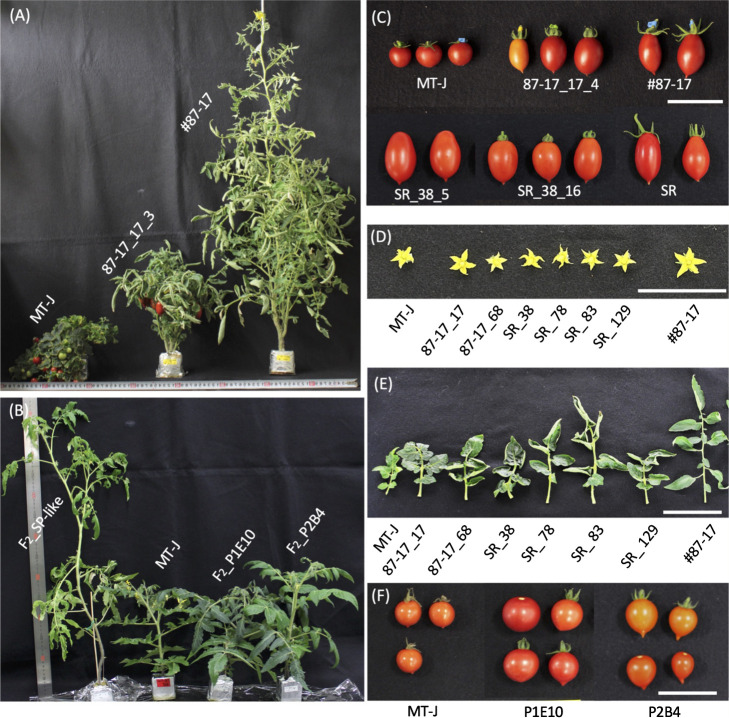
Plant and fruit phenotypes of T_1_ and F_2_ triple mutants. Some representative strains are listed. (A) Side view of a T_1_ plant 85 days after transplant (DAT). (B) Side view of F_2_ triple mutant plant 75 DAT. The control (F2_SP-like) is the F_2_ segregant that does not contain any *DWARF/SP/SlGAD3* mutation derived from Micro-Tom and #87-17. (C) Fruit appearance of T_1_ lines. (D) Flower appearance of T_1_ lines. (E) Shape of the bottom leaflet of the T_1_ lines. (F) Fruit appearance of the F_2_ triple mutant. Bars = 5 cm.

The selected genome-edited T_1_ lines were dwarfed compared to the original variety, as shown in Fig. 4A. Similarly, the selected F_2_ triple mutants were also clearly dwarfed compared to the F_2_ isolates without alleles of both genes, as shown in Fig. 4B. Fruits of T_1_ lines produced by genome editing were generally oval and plum-shaped, very similar to the characteristics of the fruit of the original cultivars #87-17 and SR and different from those of Micro-Tom (Fig. 4C). Fruits from the F_2_ triple mutant, on the other hand, were slightly more spherical and showed characteristics closer to those of Micro-Tom (Fig. 4F). The flowers of the T_1_ line were similar in size to those of Micro-Tom and slightly smaller than those of #87-17 (Fig. 4D). SR and its derivative #87-17 have curled leaf margins, which was also observed in the T_1_ line (Fig. 4E). Leaf length also tended to be shorter than in the original cultivar, but this differed among the lines (Fig. 4E). In addition, SR and #87-17 derived from it show petiolar hyponastic growth, with the petiole developing at an acute angle to the stem, the same trend seen in the T_1_ line but not seen in the F_2_ triple mutant (Fig. 4A, 4B).

### Assessment of the effects of genome editing of the DWARF and SELF-PRUNING genes on dwarfing and determination

**Fig. 5.**
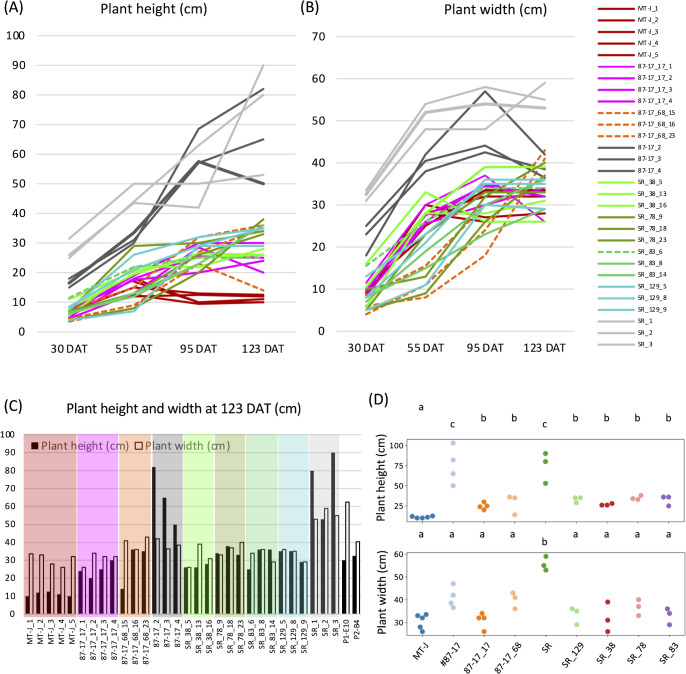
Plant height and width of T_1_ and F_2_ triple mutants. (A) Changes in plant height during cultivation. (B) Changes in plant width during cultivation. Dashed lines indicate biallelic or hetero lineages. (C) Comparison of plant length and plant width between lines including F_2_ triple mutants at 123 DAT. (D) Comparison of plant length and plant width between T_1_ lines at 123 DAT. *P* < 0.05 by the Tukey–Kramer test.

To more accurately confirm whether *DWARF* and *SELF-PRUNING* genome editing imparted dwarfism to tomato, we measured the plant height and plant width of selected T_1_ lines from approximately 30 to 123 days after transplant (DAT). The results showed that both plant height and plant width of #87-17 and SR were already greater than those of any of the T_1_ lines at 30 DAT and continued to increase until 123 DAT (Fig. 5A, dark gray, light gray). The plant width was significantly reduced at 123 DAT, partly due to the gravity-induced drooping of the long leaflets. All T_1_ lines, whether derived from #87-17 or SR, grew to a maximum plant height of less than 40 cm and were dwarfed to less than half the height of the original cultivar (Fig. 5A, 5C, Table 2). However, when compared to Micro-Tom, all T_1_ lines were also taller than Micro-Tom, especially from 95 to 123 DAT (Fig. 5A, 5C). Statistically, the plant heights of T_1_ were significantly lower in 123 DAT than in the original varieties, and some lines were rated as similar to Micro-Tom (Fig. 5D). The plant width of the T_1_ line generally maintained a trend of smaller values than the original variety and was comparable to that of Micro-Tom (Fig. 5B, 5C). Statistically, the plant width of all lines at 123 DAT was evaluated to be not significantly different, except for SR (Fig. 5D). According to the growth curve of each plant height, while the two original cultivars continued to increase, the growth of most of the T_1_ lines, including Micro-Tom, slowed down from 95 DAT to 123 DAT (Fig. 5A).

### Assessment of the effects of genome editing of the DWARF and SELF-PRUNING genes on flowering and fruit set numbers

**Fig. 6.**
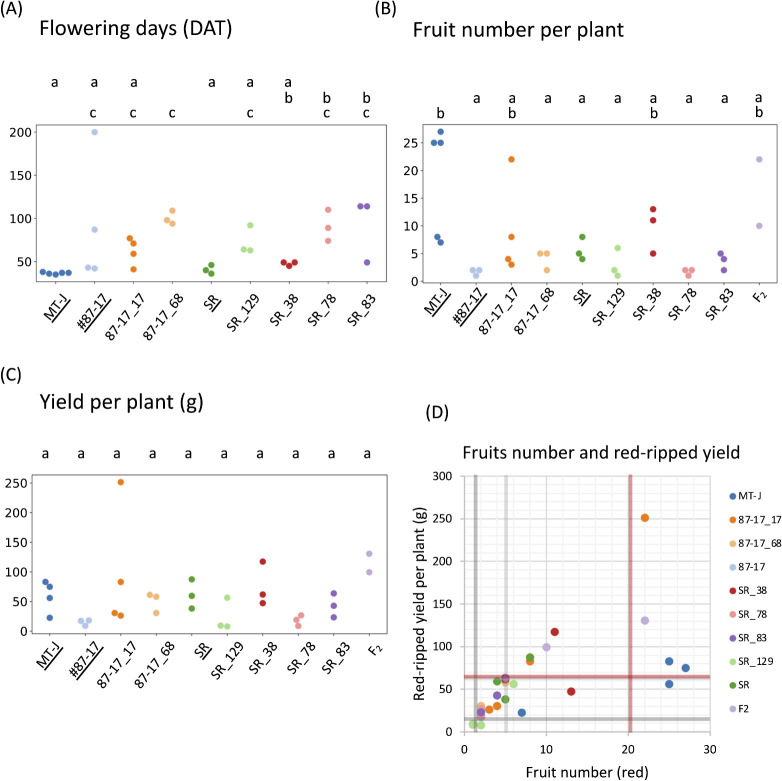
Plant phenotypes for yield-related traits in T_1_ and F_2_ triple mutants. (A) Days to first flowering in that line. (B) Fruit number per plant. (C) Yield per plant (fresh weight). (D) Relationship between fruit number and yield for each line. Red lines show the average of MT-J, dark gray lines show the average of #87-17 and pale gray lines show the average of SR. The names for control on graphs (MT-J, #87-17 and SR) are underlined. *P* < 0.05 by the Tukey–Kramer test.

Micro-Tom can grow normally even under fluorescent light and has a short period from sowing to fruiting (Shikata and Ezura 2016). Thus, under the cultivation conditions used in this report, the number of days from transplantation to flowering of the first flower was measured. As a result, T_1_ lines 87-17_68 (mean 100.3 DAT), SR_78 (mean 91 DAT) and SR_83 (mean 92.3 DAT) had significantly longer days until flowering than Micro-Tom, which bloomed at an average of 36.6 DAT. It was shown to be statistically similar to #87-17 (mean 57.3 DAT) (Fig. 6A, Table 2). In addition, 87-17_17 (mean 62 DAT), SR_129 (mean 73 DAT), and SR_38 (mean 47.7 DAT) did not have a statistically significant difference from Micro-Tom, but there was still a tendency for the number of days from transplantation to flowering of the first flower to increase (Fig. 6A, Table 2). We then counted the number of fruits set per plant. As a result, compared with Micro-Tom, for which an average of 18.4 fruits was recorded, almost all the T_1_ lines except 87-17_17 (mean 9.3) and SR_38 (mean 9.7) decreased in number of fruits, and the number of fruits set were at the same level as in #87-17 and SR (Fig. 6B, Table 2). On the other hand, the average number of fruits borne by the two F_2_ triple mutants was 16, which was statistically similar to that of both Micro-Tom and the original cultivar (Fig. 6B).

The number of fruits set in the T_1_ line was as small as that of the original cultivar, but the fruit size was larger than that of Micro-Tom, as shown in Fig. 4C. Therefore, the red-ripe fruit yields per plant were calculated and compared. Although there was variation in each line, there were no statistically significant differences (Fig. 6C). Fig. 6D shows the relationship between the number of fruit set on the horizontal axis and the yield of ripe red fruit on the vertical axis, plotted in a scatter diagram. According to this, most of the T_1_ lines, with the exception of SR_129, exceeded at least the average fruit setting and red-ripe fruit yield of #87-17, and the fruit setting and red-ripe fruit yield of 87-17_17, some SR_38, and the F2 triple mutant also exceeded the average of SR (Fig. 6D). In particular, 87-17_17_3 exceeded the average yield of Micro-Tom, and it was shown that even T_1_ lines with the same indel had variations among individual plants (Fig. 6D).

### Assessment of fruit traits of genome-edited T_1_ lines

**Fig. 7.**
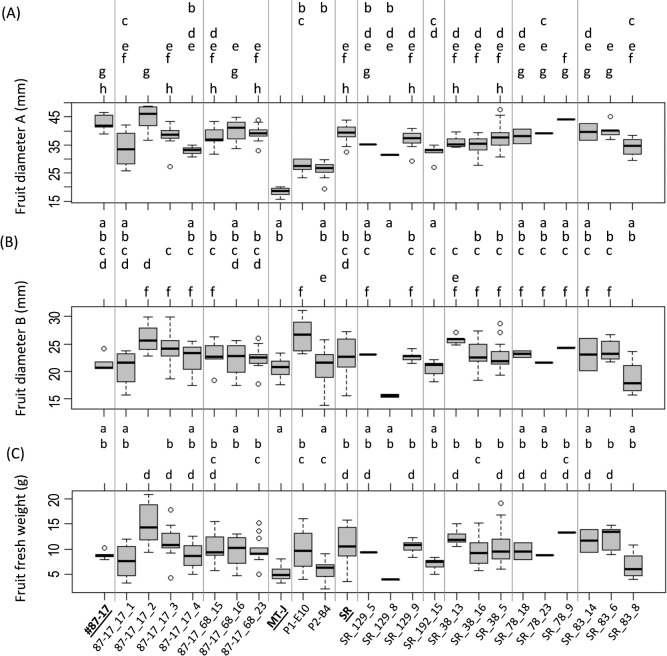
Fruit phenotypes in T_1_ and F_2_ triple mutants. (A) Maximum vertical diameter of fruit: A. (B) Maximum horizontal diameter of fruit: B. (C) Single fruit fresh weight. The names for the control on the graphs (MT-J, #87-17 and SR) are underlined. *P* < 0.05 by the Tukey–Kramer test.

The fruit of #87-17 and SR is plum-shaped and medium-sized (Fig. 4C), while Micro-Tom’s fruit shape is spherical and small (Fig. 4C, 4F). Therefore, to evaluate the effects of *DWARF* and *SELF-PRUNING* genome editing on fruit shape, we measured the maximum vertical diameter of fruit (A), the maximum horizontal diameter (B) and the single fruit fresh weight. The results showed that the maximum longitudinal diameter A was predominantly larger in all T_1_ lines and F_2_ triple mutants than in Micro-Tom. In addition, all T_1_ lines were similar to the original cultivar #87-17 or SR (Fig. 7A). On the other hand, the maximum transverse diameter of B was not significantly different from that of Micro-Tom, even in some of the T_1_ lines and the F_2_ triple mutant P2-B4, probably because there was no significant difference between the values of #87-17 and Micro-Tom to begin with (Fig. 7B). However, none of the T_1_ lines, with the exception of SR_129_8, showed at least significant differences from the original variety (Fig. 7B). The fresh weight per fruit was not significantly different from that of Micro-Tom for #87-17 itself, but that of the T_1_ line derived from #87-17 was similar to or significantly higher than that of #87-17 (Fig. 7C). On the other hand, SR had significantly higher values than Micro-Tom, and most of the SR-derived T_1_ lines also had similar values to SR (Fig. 7C). In the fruit fresh weight of F_2_ triple mutant, P1-E10 was predominantly different from Micro-Tom but similar to #87-17 and SR, and showed higher values (Fig. 7C). In contrast, P2-B4 showed similar trends to Micro-Tom (Fig. 7C).

**Fig. 8.**
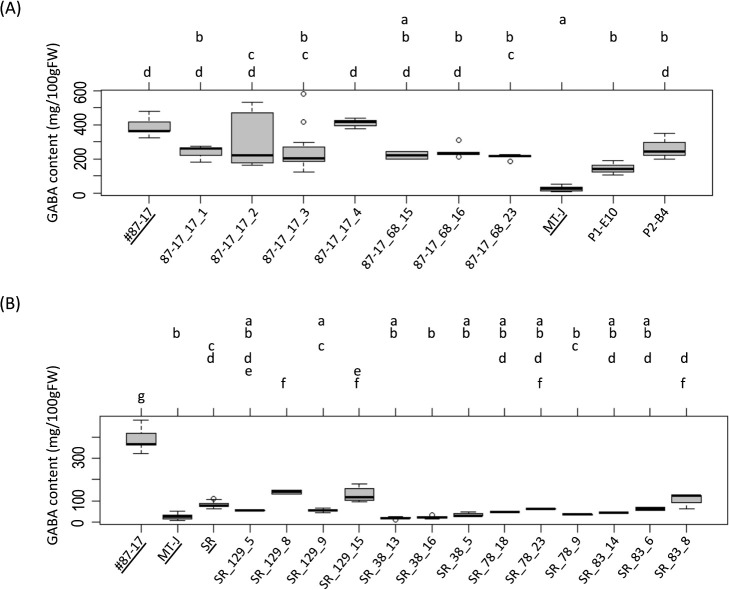
Fruit GABA contents in T_1_ and F_2_ triple mutants. (A) Comparison of fruit GABA contents in #87-17-derived high-GABA T_1_ lines and F_2_ triple mutants. (B) Comparison of fruit GABA contents in SR-derived normal-GABA T_1_ lines. The names for control on graphs (MT-J, #87-17 and SR) are underlined. *P* < 0.05 by the Tukey–Kramer test.

Next, the GABA content of the fruit was measured to evaluate the effect of *DWARF* and *SELF-PRUNING* genome editing on the high GABA content in the fruit as given by the active *SlGAD3* derived from the #87-17. Note that the fruit samples used were those with seeds removed, so the values shown do not reflect the amount of GABA contained in the seeds. Except few lines (P1-E10, 87-17_17_3 and 87-17_68_23), the GABA content of the #87-17 derived T_1_ lines and F_2_ triple mutants having the #87-17 type *SlGAD3* gene lacking the autoinhibitory domain were retained GABA content levels comparable to those of the original variety #87-17 (Fig. 8A). The SR derived T_1_ lines, SR_129_5, SR_129_9, SR_78_18, SR_78_23, SR_78_9, SR_83_14, SR_83_6 and SR_83_8 retained similar GABA contents to the original cultivar, while other SR derived T_1_ lines, SR_38_13, SR_38_16 and SR_38_5 had significantly lower GABA contents than the original cultivar (Fig. 8B). Additionally, SR_129_8 and SR_129_15 have significantly higher GABA contents than SR despite having same wild-type GAD3.

### PCA of T_1_ lines and F_2_-triple mutants

**Fig. 9.**
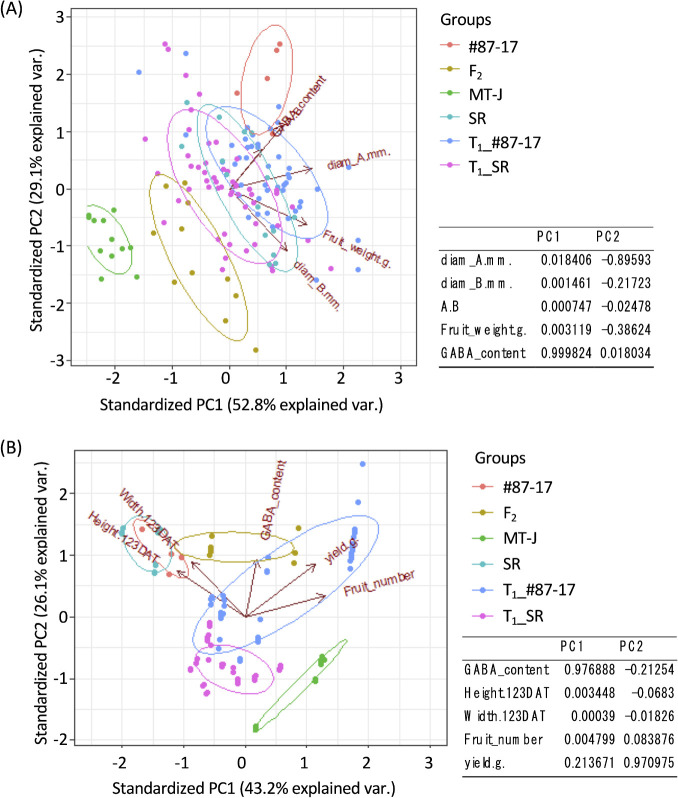
Differential phenotype analysis. (A) PCA of only the fruit phenotype in each line. (B) PCA of the main plant phenotype in each line.

To comprehensively evaluate the traits of the T_1_ lines and F_2_ triple mutants measured up to this point, principal component analysis was performed for the target traits and for the main traits related to yield. First, principal component analysis was performed only for fruit-related traits (maximum longitudinal diameter: A, maximum transverse diameter: B, aspect ratio: A, B, fruit fresh weight, and GABA content). The results showed that PC1 and PC2 explained 81.9% of the data, with GABA content being the factor contributing most to PC1 and maximum longitudinal diameter being the factor contributing most to PC2 (Fig. 9A). The original genome-edited variety and one parent of the cross, #87-17, were distributed in the upper right-most part of the plot, while Micro-Tom, a dwarfing indicator containing mutations in two genes targeted by genome editing, was independently distributed in the lower left-most part of the plot (Fig. 9A). SR, another original variety for genome editing, was distributed slightly below #87-17, and T_1_ lines derived from #87-17 (T_1__#87-17) and SR-derived T_1_ lines (T_1__SR) overlapped this group (Fig. 9A). On the other hand, the F_2_ triple mutants were distributed mostly near the middle of both parental cultivars, although they overlapped partially with the distribution of T_1__SR, suggesting that they inherited traits, especially GABA content and A/B ratio, intermediate between #87-17 and Micro-Tom (Fig. 9A). Both T_1_ lines and SR and F_2_ triple mutant groups varied in maximum lateral diameter: B and fresh fruit weight factors (Fig. 9A).

Next, principal component analysis was conducted on the main traits of the entire plant body related to yield, including plant height, which was the target trait for this study (height.123DAT, width.123DAT, fruit_number, fresh yield.g., and GABA content). The results showed that PC1 and PC2 explained 69.3% of the data, with GABA content being the factor contributing most to PC1 and fresh yield being the factor contributing most to PC2 (Fig. 9A). The original genome-edited variety and one parent of the cross, #87-17, were distributed in the upper left-most part of the plot along with SR, while Micro-Tom, a dwarfing indicator containing mutations in two genome-edited target genes, was independently distributed in the lower right-most part of the plot (Fig. 9B). Both T_1__#87-17 and T_1__SR did not overlap with Micro-Tom but were distributed closer to Micro-Tom compared to the F_2_ triple mutant group (Fig. 9B). Conversely, the F_2_ triple mutant was distributed close to the original variety #87-17 and SR groups; the T_1__#87-17 line was variable with respect to fresh yield, while T_1__SR showed relatively little variation (Fig. 9B).

## Discussion

### Genome editing of the DWARF and SELF-PRUNING genes resulted in dwarfing and determination of #87-17 and SR

All T_1_ lines genome-edited for the *DWARF* and *SELF-PRUNING* genes by CRISPR–Cas9 showed more than half the dwarfing and centrality of the original varieties. This indicates that *DWARF* and *SELF-PRUNING* genome editing can impart dwarfing traits to the indeterminate cultivars that have medium-sized fruit (#87-17) and its original variety SR. Plant width also tended to be smaller than that of the original variety at all stages up to 123 DAT. The reason why the plant width at 123 DAT was similar to that of #87-17 is that the leaf blade of the original variety was elongated as it grew, which caused it to sag due to gravity resistance. This is thought to be because the leaf blades of the original cultivar were not able to withstand gravity and drooped down as they grew. This inference is also supported by the plant shape of #87-17 in Fig. 4A. On the other hand, the plant height of the T_1_ lines in particular is still larger than that of micro-Tom, the original mutant of *DWARF* and *SELF-PRUNING*. This suggests, as previously mentioned (Marti *et al.* 2006, Meissner *et al.* 1997), that another gene other than *DWARF*, such as *miniature*, is involved in the dwarf trait of Micro-Tom.

However, the maximum plant height currently recommended for plant factory cultivation is approximately 30 cm (Kozai 2013), and all of the T_1_ lines produced in this study met this target (Fig. 5D). The average plant width was also similar to that of Micro-Tom (Fig. 5D), indicating that more plantlets can be grown on a limited number of growing racks.

Similar to the *d* allele of the *DWARF* gene, the *sp* allele of *SELF-PRUNING* gene of Micro-Tom is also recessive (Pnueli *et al.* 1998). However, among the T_1_ lines showing dwarf traits produced in this study, the heterozygous *sp* mutations (87-17_68_23 and SR_38_5) had no effect on dwarfing, and none of them showed a indeterminate phenotype (Fig. 5A, 5C). It also did not seem to confer other phenotypic differences compared to other homozygous lines or mutant lines with early translation termination (Figs. 6–8). Whether this is due to molecular genetic effects or synergistic effects with the characteristics of the original SR variety remains to be verified and will require further study.

### The dwarfed T_1_ lines produced by genome editing retained the fruit traits of the original variety

The original cultivars #87-17 and SR used in this study are both plum-shaped, medium-sized fruit-forming cultivars. Both T_1_ lines used in this study retained this trait almost remarkably. This suggests that the genome editing of the *DWARF* and *SELF-PRUNING* genes performed in this study did not affect the function of several important genes such as *SUN*, *LC*, *OVATE*, and *FAS* that contribute to fruit traits (Maurya *et al.* 2021, Rodríguez *et al.* 2011).

Conversely, the F_2_ triple mutant produced by these crosses showed dwarf phenotype as well as genome edited T_1_ lines (Figs. 4B, 5C), but they formed more round fruits which were intermediate between #87-17 and Micro-Tom (Figs. 4F, 9A). This reflects the fact that, in contrast to the T_1_ line, many genes involved in fruit formation were brought over from Micro-Tom. To eliminate these factors and to achieve the original fruit quality of #87-17, it is necessary to perform several backcrosses with #87-17 in the future. In this respect, it was confirmed once again that the targeted traits could be more efficiently conferred by genome editing (Fig. 9B), and at the same time, the nontarget traits of the original cultivar were more efficiently retained (Fig. 9A) compared to the conventional method of crossbreeding.

One concern is that GABA content was reduced in some of the dwarfed T_1_ lines (Fig. 8). In particular, there were many SR-derived T_1_ lines with reduced GABA content. At this stage, there is no clear link between reduced GABA content in these T_1_ lines and dysfunction of the *DWARF* or *SELF-PRUNING* genes. On the other hand, GABA content is known to be altered to some extent by environmental conditions and biotic and abiotic stresses (Ham *et al.* 2012, Kim *et al.* 2013). Therefore, we cannot exclude the possibility that the loss of function of the *DWARF* gene CYP85A1, which is involved in brassinosteroid biosynthesis, may have significantly reduced the brassinosteroid content in the plant and affected GABA biosynthesis. The interindividual differences in the degree of reduction in GABA content cannot be explained solely by genetic background.

### The 1- and 2-amino acid deletion mutations of DWARF generated in this study may cause DWARF dysfunction

As reported previously (Bishop *et al.* 1996, Marti *et al.* 2006), the *d* allele, a loss-of-function mutation in *DWARF*, is recessive. However, some of the T_1_ lines showing the dwarf trait that we have generated have biallelic mutations (87-17_68_15, 16, 23, SR_83_6) that are deficient in the DWARF protein (CYP85A) by only one or two amino acids and early translation termination. However, all of these lines showed dwarf traits comparable to those of other early translation-terminating mutant lines (Fig. 5C). This suggests that these 1- and 2-amino acid deletion mutations also cause DWARF dysfunction. Since Y216 and H217 are part of the α-helix spatially adjacent to the heme-binding domain of the active center (Supplemental Figs. 2, 3), it can be inferred that the loss of these amino acids may impair the protein 3D structure related to enzyme function. These possibilities need to be verified in detail in the future.

### Estimation of fresh fruit yield per area when the T_1_ line produced in this study is applied in a plant factory

Based on the average space occupied by the T_1_ lines and the fresh yield per plant, we calculated the estimated fresh fruit yield per unit area (m^2^) of a growing shelf when the T_1_ lines produced in this study were applied in a plant factory. The results are summarized in Table 3. The best average fresh fruit yield per plant among the T_1_ lines produced in this study was the 87-17_17 line, and when that line was used for plant factory cultivation, the number of plants per unit area was 10.4 plants/m^2^, almost the same level as that of Micro-Tom (Table 3). Furthermore, the estimated fresh fruit yield per unit area was 1017.7 g/m^2^ (Table 3), which was approximately 10 times higher than that of the original cultivar #87-17 and 5 times higher than that of SR. Conversely, the line with the lowest average fresh fruit yield per plant was SR_78, and when that line was used for plant factory cultivation, the number of plants grown per unit area was 7.4 plants/m^2,^ and the estimated fresh fruit yield per unit area was 135.3 g/m^2^ (Table 3). Furthermore, when the vertical exclusive area is taken into account, each T_1_ line is less than 40 cm (Fig. 5), so it is calculated that twice as many plants of the original variety can be grown within a 1 m height space. Therefore, depending on the specifications of the plant factory used, it is possible to further increase the efficiency of cultivation by stacking several 40–50 cm tall growing racks, which is difficult to do with the original variety.

The number of fruit set per plant is strongly influenced not only by genetic factors but also by environmental factors (Huat *et al.* 2013). The T_1_ lines obtained in this study generally tended to have a later flowering date than the original cultivar (Fig. 6A). Tomato flowering is also strongly affected by light intensity in the growing environment. These negative changes in cultivation may possibly be related to changes in grass type associated with dwarfing. Dwarfed T_1_ lines tend to have leaves concentrated in the center, as shown in Fig. 4A, coupled with the leaf characteristics of the original SR varieties, which are formed at an acute angle to the stem due to the downward extension of the petiole. This concentration of leaves increases the degree of leaf-to-leaf overlap and may adversely affect photosynthetic efficiency. Unfortunately, it is not possible to draw firm conclusions at this stage as to which of these influences is responsible for the variation in fresh fruit yield per plant in this study. Phenotypic studies after the T_2_ generation and evaluations in cultivation environments with varying environmental factors are needed to clarify this, but at least according to the various findings reported to date, further improvements are expected to be possible. As one of the options, further genetic modification by genome editing of genes related to the number of fruit set and flowering days, such as *SlSP5G* and *SlIAA9*, and genes inducing monogenic results (Ariizumi *et al.* 2013, Soyk *et al.* 2017), for example, can be considered candidates.

### Summary

In this paper, we showed that genome editing of two genes, *DWARF* and *SELF-PRUNING*, by CRISPR–Cas9 imparts target traits to cultivars more efficiently and effectively than conventional breeding. The resulting genome-edited T_1_ lines are expected to be able to be grown at densities comparable to those of Micro-Tom, in which case the highest yielding lines can be selected to produce fresh fruit yields approximately five to ten times higher than those of the original cultivar. Naturally, improvement efforts will be required to maximize the efficiency, including continued optimization of the growing environment and further genome editing of favorable mutations and pyramiding of mutations.

## Author Contribution Statement

AN and HE conceived this paper. AN provided the data, and AN and HE wrote the manuscript. All the authors have read and approved the final manuscript.

## Supplementary Material

Supplemental Figures

Supplemental Table

## Figures and Tables

**Table 1.** List of genotypes and indels of each T_1_ lines and the original cultivars

Type	Line name	Genotype		
		*D*: Indel	*SP*: Indel	*SlGAD3*
Wild type	MT-J_1	*d*	*sp*	*gad3*
Wild type	MT-J_2	*d*	*sp*	*gad3*
Wild type	MT-J_3	*d*	*sp*	*gad3*
Wild type	MT-J_4	*d*	*sp*	*gad3*
Wild type	MT-J_5	*d*	*sp*	*gad3*
GE_T1	87-17_17_1	+1	–1	*GAD3*
GE_T1	87-17_17_2	+1	–1	*GAD3*
GE_T1	87-17_17_3	+1	–1	*GAD3*
GE_T1	87-17_17_4	+1	–1	*GAD3*
GE_T1	87-17_68_15	–2/–3	–11/+1	*GAD3*
GE_T1	87-17_68_16	–2/–3	–11	*GAD3*
GE_T1	87-17_68_23	–2/–3	–11/0	*GAD3*
GE_T4	87-17_2	*D*	*SP*	*GAD3*
GE_T4	87-17_3	*D*	*SP*	*GAD3*
GE_T4	87-17_4	*D*	*SP*	*GAD3*
GE_T1	SR_38_5	+1	0/–6	*gad3*
GE_T1	SR_38_13	–1	+1	*gad3*
GE_T1	SR_38_16	–1	+1	*gad3*
GE_T1	SR_78_9	+1	–1/+1	*gad3*
GE_T1	SR_78_18	+1	–1/+1	*gad3*
GE_T1	SR_78_23	+1	+1	*gad3*
GE_T1	SR_83_6	–1/–6	+1	*gad3*
GE_T1	SR_83_8	+1	+1	*gad3*
GE_T1	SR_83_14	+1	+1	*gad3*
GE_T1	SR_129_5	+1	+1	*gad3*
GE_T1	SR_129_8	+1	+1	*gad3*
GE_T1	SR_129_9	–1	+1	*gad3*
GE_T1	SR_192_2	+1	+1	*gad3*
GE_T1	SR_192_15	0/–6	+1	*gad3*
Wild type	SR_1	*D*	*SP*	*gad3*
Wild type	SR_2	*D*	*SP*	*gad3*
Wild type	SR_3	*D*	*SP*	*gad3*
F_2__triple_mutant	P1-E10	*d*	*sp*	*GAD3*
F_2__triple_mutant	P2-B4	*d*	*sp*	*GAD3*

MT-J, Micro-Tom Japan; SR, sicilian rouge; GE, Genome editing; *D*, *DWARF*; *SP*, *SELF-PRUNING*.

**Table 2.** Summary of phenotypes associated with dwarfism and yield for each T_1_ line

Line name	Genotype/Indel		Plant height (cm) Ave.					Plant width (cm) Ave.				Flowering days (DAT) Ave.	Fruit number (riped) Ave.	Red-riped yield per plant (g) Ave.
	*DWARF*	*SELF-PRUNING*	30 DAT	55 DAT	95 DAT	123 DAT		30 DAT	55 DAT	95 DAT	123 DAT			
MT-J	*d*	*sp*	6.8 ± 0.2	14.9 ± 2.4	11.0 ± 1.4	11.1 ± 1.0		9.7 ± 0.6	26.8 ± 2.5	30.3 ± 3.2	30.5 ± 3.0	36.6 ± 1.0	18.4 ± 8.9	59.1 ± 23.3
87-17	*D*	*SP*	16.5 ± 1.2	31.5 ± 1.5	61.0 ± 5.3	65.7 ± 13.1		22.0 ± 2.9	40.2 ± 1.6	47.9 ± 6.5	39.0 ± 2.3	57.3 ± 21.0	1.7 ± 0.5	14.8 ± 4.1
SR	*D*	*SP*	27.5 ± 2.9	45.8 ± 3.0	51.7 ± 8.7	74.3 ± 15.6		32.3 ± 1.0	51.3 ± 2.5	53.3 ± 4.1	55.7 ± 2.5	40.7 ± 4.1	5.7 ± 1.7	61.7 ± 20.1
87-17_17	+1	–1	5.9 ± 1.2	16.9 ± 2.3	26.0 ± 3.8	24.8 ± 3.6		9.9 ± 1.1	27.3 ± 1.8	34.1 ± 2.6	31.0 ± 3.0	62.0 ± 13.7	9.3 ± 7.6	97.8 ± 91.5
87-17_68	–2/–3	–11 –11/+1 –11/0	5.2 ± 1.7	10.7 ± 3.1	27.3 ± 3.7	28.3 ± 10.1		6.2 ± 1.8	11.5 ± 3.1	24.0 ± 4.9	40.0 ± 2.9	100.3 ± 6.3	4.0 ± 1.4	49.9 ± 13.7
SR_38	+1 –1	0/–6 +1	8.2 ± 2.2	20.7 ± 1.0	24.5 ± 1.9	26.7 ± 0.9		10.7 ± 4.2	29.7 ± 2.4	31.0 ± 5.7	32.0 ± 5.4	47.7 ± 1.9	9.7 ± 3.4	75.5 ± 30.2
SR_78	+1	–1/+1	6.0 ± 1.4	16.7 ± 9.0	25.3 ± 4.1	35.0 ± 2.2		6.7 ± 2.4	15.0 ± 5.9	30.3 ± 3.1	36.7 ± 2.9	91.0 ± 14.8	1.7 ± 0.5	18.2 ± 7.3
SR_83	–1/–6 +1	+1	8.0 ± 2.5	15.7 ± 4.5	23.7 ± 0.9	32.3 ± 5.2		10.2 ± 3.8	20.0 ± 4.5	29.0 ± 4.5	33.0 ± 2.9	92.3 ± 30.6	3.7 ± 1.2	43.3 ± 16.5
SR_129	+1 –1	+1	6.7 ± 1.9	18.0 ± 8.0	30.0 ± 1.4	33.0 ± 2.8		8.7 ± 3.3	18.3 ± 5.2	33.7 ± 2.6	33.3 ± 3.1	73.0 ± 13.4	3.0 ± 2.2	24.5 ± 22.6

MT-J, Micro-Tom Japan; SR, sicilian rouge; *D*, *DWARF*; *SP*, *SELF-PRUNING*, DAT, days after transplant.

**Table 3.** Estimation of fresh yield per area when the T_1_ line produced in this study is applied in a plant factory

Line name	Genotype/Indel			Plant height (cm) Ave.	Plant width (cm) Ave.	Red-riped yield per plant (g) Ave.	Predicted fresh yield per unit area (g/m^2^)	Predicted plant number per unit area (plant/m^2^)
	*DWARF*	*SELF-PRUNING*		123 DAT	123 DAT			
MT-J	*d*	*sp*		11.1 ± 1.0	30.5 ± 3.0	59.1 ± 23.3	635.4	10.7
87-17	*D*	*SP*		65.7 ± 13.1	39.0 ± 2.3	14.8 ± 4.1	97.2	6.6
SR	*D*	*SP*		74.3 ± 15.6	55.7 ± 2.5	61.7 ± 20.1	199.2	3.2
87-17_17	+1	–1		24.8 ± 3.6	31.0 ± 3.0	97.8 ± 91.5	1017.7	10.4
87-17_68	–2/–3	–11 –11/+1 –11/0		28.3 ± 10.1	40.0 ± 2.9	49.9 ± 13.7	312.1	6.3
SR_38	+1 –1	0/–6 +1		26.7 ± 0.9	32.0 ± 5.4	75.5 ± 30.2	736.9	9.8
SR_78	+1	–1/+1		35.0 ± 2.2	36.7 ± 2.9	18.2 ± 7.3	135.3	7.4
SR_83	–1/–6 +1	+1		32.3 ± 5.2	33.0 ± 2.9	43.3 ± 16.5	397.3	9.2
SR_129	+1 –1	+1		33.0 ± 2.8	33.3 ± 3.1	24.5 ± 22.6	220.7	9.0

MT-J, Micro-Tom Japan; SR, sicilian rouge; *D*, *DWARF*; *SP*, *SELF-PRUNING*, DAT, days after transplant.
