# Supplementary material for: Genome editing of DWARF and SELF-PRUNING rapidly confers traits suitable for plant factories while retaining useful traits in tomato
Source: Breed Sci. 2024 Apr 4;74(1):59–72. doi: 10.1270/jsbbs.23063 (PMC11375428; doi:10.1270/jsbbs.23063)
Supplement: Supplementary file 1 — Supplemental Figures [file 74_059_s1.pdf]

Supplemental figure 1

Nagamine and Ezura/Breeding Science
doi: 10.1270/jsbbs.23063

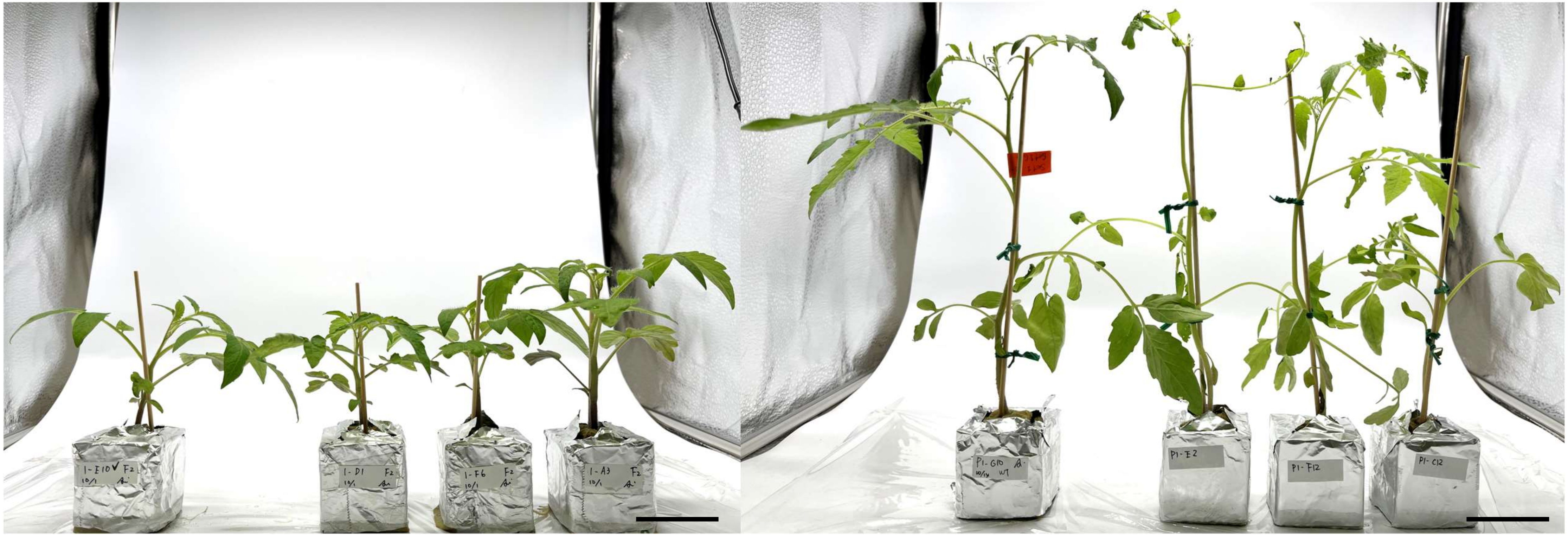

dd/ss *dd/SS* *DD/SS* *DD/ss*

Supplemental figure 1 F2 segregants from Micro-Tom x #87-17 crossing. Each genotype for *DWARF* (*D*) and *SELF-PRUNING* (*S*) which were checked by each dCAPs are labeled at bottom of each F2 group. Bars=5 cm

Supplemental figure 2

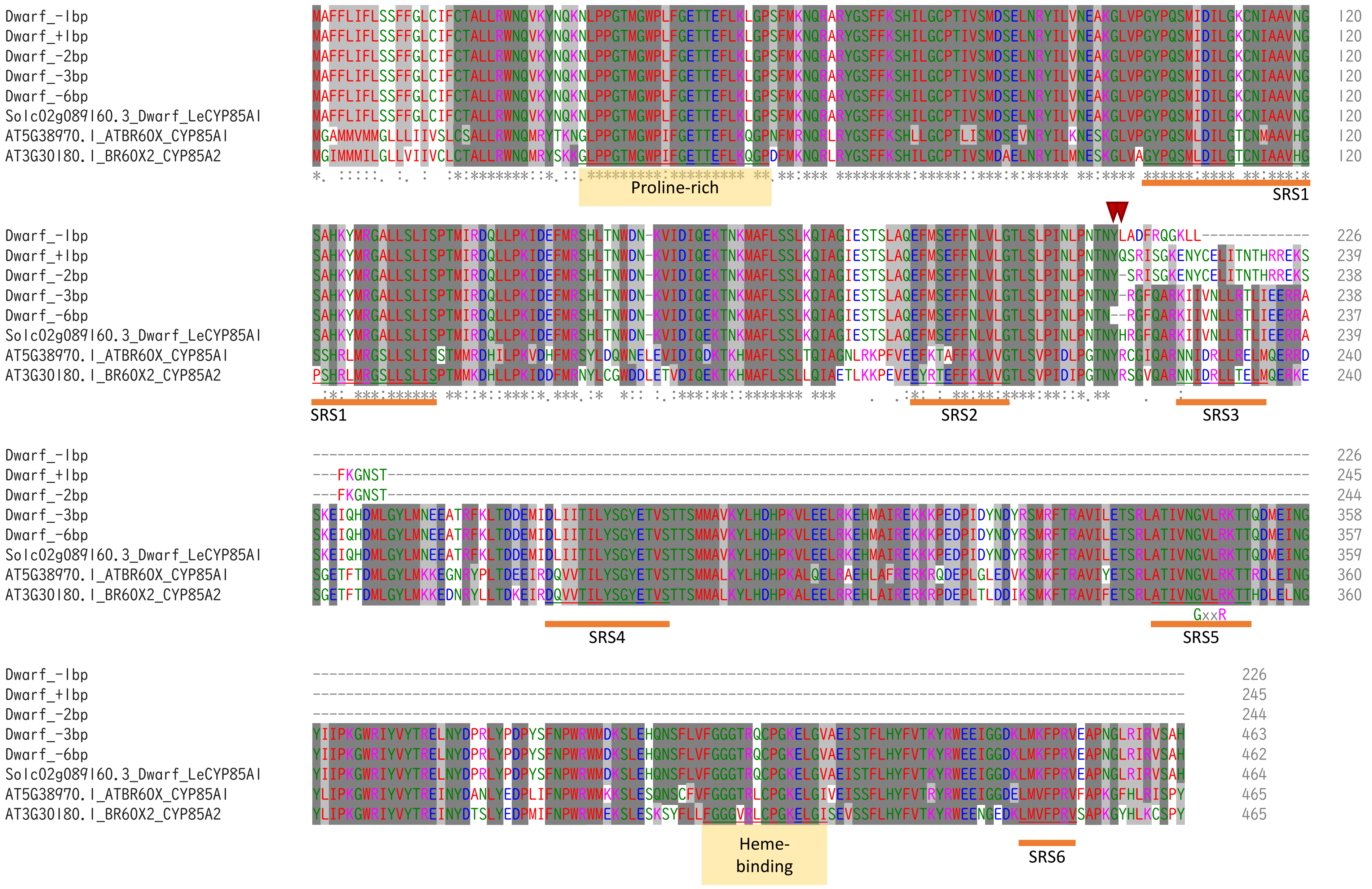

Supplemental figure 2 Each *DWARF* gene from tomato and Arabidopsis thaliana amino acid alignment. For tomato gene, each indel derived truncated protein sequences are also aligned. Gray highlight and light gray highlight shows common or similar amino acid in all, respectively.

Supplemental figure 3

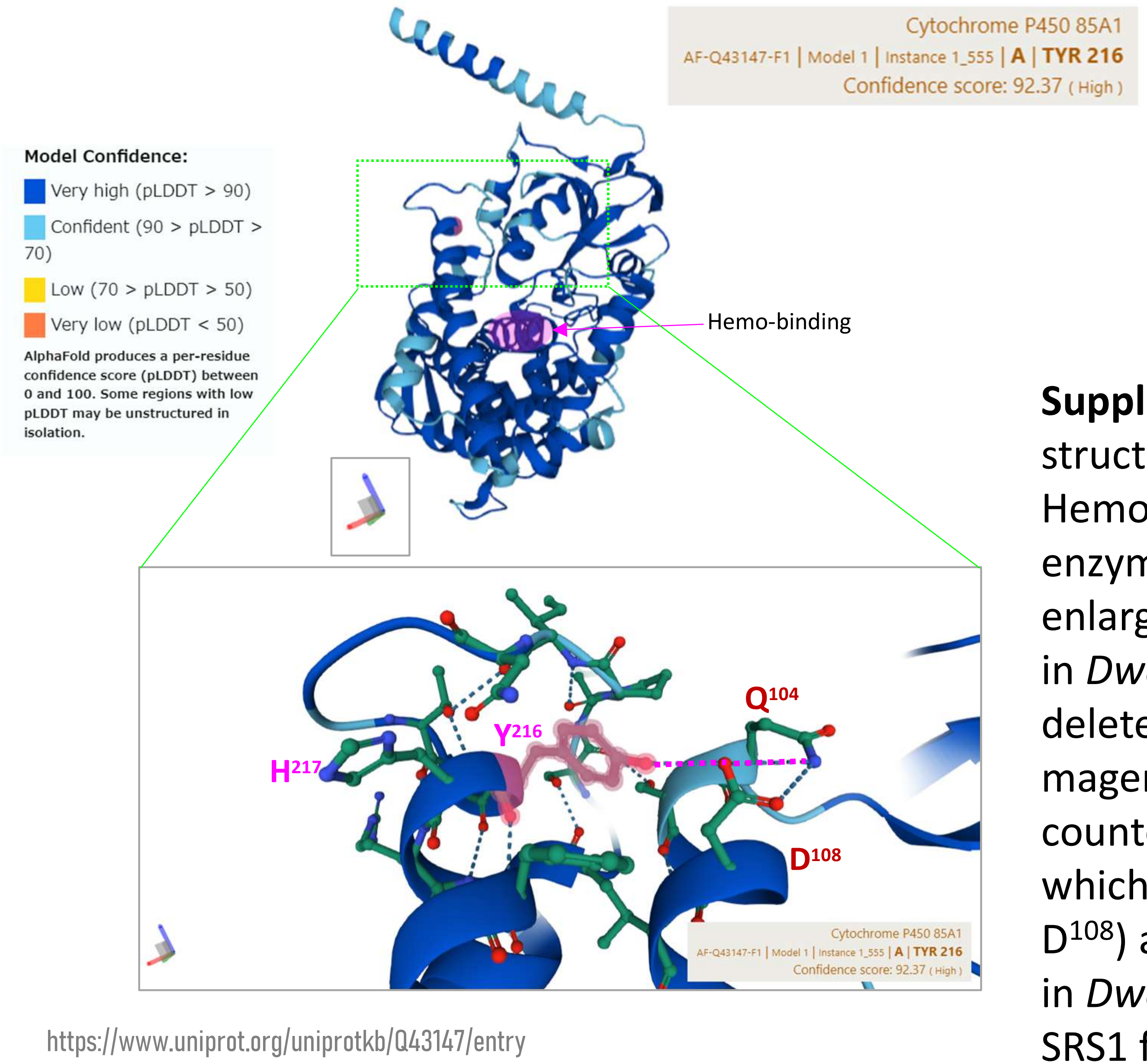

Supplemental figure 3 DWARF protein 3D structure which is referred from Uniprot DB. Hemo-binding site which is important for enzymatic activity is shown in magenta. In enlarged image, both H^{217} which is deleted in *Dwarf*-3 bp,-6 bp and Y^{216} which is deleted in *Dwarf*-6 bp are shown in magenta letter and the putative counterparts of hydrogen-bonds for Y^{216} which highlighted in magenta (Q^{104} and D^{108}) are shown in red. Y^{216} which is deleted in *Dwarf*-6 bp will bind with Q^{104} and D^{108} in SRS1 for substrate recognition via hydrogen-bonds.
